# История института высшего и дополнительного профессионального образования ГНЦ РФ ФГБУ «НМИЦ эндокринологии» Минздрава России

**DOI:** 10.14341/probl13470

**Published:** 2024-06-04

**Authors:** С. В. Сумина, Е. А. Пигарова, Л. К. Дзеранова, А. Г. Кузьмин

**Affiliations:** Национальный медицинский исследовательский центр эндокринологии; Национальный медицинский исследовательский центр эндокринологии; Национальный медицинский исследовательский центр эндокринологии; Национальный медицинский исследовательский центр эндокринологии

**Keywords:** ординатура, образование, эндокринология

## Abstract

ГНЦ РФ ФГБУ «НМИЦ эндокринологии» Минздрава России гордится не только своими достижениями в области персонализированного подхода к обследованию и лечению пациентов в соответствии с принципами доказательной медицины, использованием современных технологий диагностики и лечения, но и богатейшей историей подготовки научных и лечебных кадров. Центр на протяжении многих лет привлекает лучших и наиболее талантливых выпускников высших медицинских учреждений, становясь alma mater для молодых врачей — эндокринологов, детских эндокринологов и диетологов. Специалисты, выпускники НМИЦ эндокринологии, вносят значительный вклад в развитие медицины, проводя исследования, создавая инновационные методы лечения и помогая пациентам не только в нашей стране, но и за ее пределами. Однако ГНЦ РФ ФГБУ «НМИЦ эндокринологии» знаменит не только своим качественным и всесторонним обучением, но и опытным, преданным своему делу преподавательским составом, дающим обучающимся уникальные возможности для профессионального роста. Целью данной статьи является отражение наиболее важных вех в развитии образования в НМИЦ эндокринологии, которое начало свое становление более века назад, в тяжелейший для нашей страны переходный период.

## Abstract (en)

The Endocrinology Reserch Centre is proud not only of its achievements in the area of personalized approach to the examination and treatment of patients in accordance with the principles of evidence-based medicine, the use of modern diagnostic and treatment technologies, but also of its rich history of training scientific and medical personnel. For many years the Center has been attracting the best and most talented graduates of higher medical institutions, becoming the “alma mater” for young doctors - endocrinologists, pediatric endocrinologists and nutritionists. Specialists, graduates of NMRC, make a significant contribution to the development of medicine, conducting research, creating innovative methods of treatment and helping patients not only in our country, but also abroad. However, the State Research Center of the Russian Federation FGBU «NMRC Endocrinology» is famous not only for its high-quality and comprehensive training, but also for its experienced, dedicated teaching staff, giving students unique opportunities for professional growth. The purpose of this article is to reflect the most important milestones in the development of education at NMIC Endocrinology, which began its formation more than a century ago, during the most difficult period of transition for our country.

## Исторический очерк

История образовательных программ по эндокринологии начинается с 1896 г. и связана с именем основателя русской клинической эндокринологии Василия Дмитриевича Шервинского. Именно благодаря его деятельности были организованы лекции по эндокринным заболеваниям для терапевтов и студентов высших медицинских учреждений. В своих лекциях Василий Дмитриевич первым в нашей стране изложил клинику базедовой болезни, микседемы, аддисоновой болезни, нарушений роста, гипофизарных заболеваний и др. В лекционном материале он подчеркивал необходимость комплексного применения различных методов лечения: диеты, медикаментозной терапии, хирургического вмешательства, а также большое значение придавал личной и общественной гигиене в предупреждении заболеваний и лечении больных, которое называл «общественным лечением». Для улучшения педагогического процесса Шервинский ввел в практику демонстрацию музейных анатомических и патоморфологических препаратов. В 1929 г., совместно с отечественным патологом Гавриилом Петровичем Сахаровым, Василий Дмитриевич выпустил первое фундаментальное отечественное руководство по клинический эндокринологии «Основы эндокринологии», которое стало настольной книгой для подготовки поколений эндокринологов и по многим параметрам актуально до сих пор. В последующем, благодаря настойчивости и энергичности В.Д. Шервинского, в 1933 г. организовываются курсы подготовки врачей-терапевтов по эндокринологии на базе Центрального института усовершенствования врачей, что в дальнейшем стало основой для создания кафедры эндокринологии.

## Становление ординатуры

Ординатура, как считается, берет свое начало с предложения 1843 г. профессоров А.И. Овера и А.И. Поля из клиник факультетской терапии и госпитальной хирургии, соответственно, медицинского факультета Московского университета. Согласно этому предложению, рекомендовалось назначать помощниками ассистентов и адъюнктов госпитальных клиник лучших студентов последнего курса. Эти обучающиеся должны были жить в больничном здании и несколько раз в день навещать больных, которых вели эти ассистенты, в палатах и тем самым быстрее знакомиться с практическими предметами. Однако в таком виде ординатура не была реализована, но в качестве прообраза современной ординатуры организована 2-годичная конкурсная ассистентура.

Ординатура же, как форма подготовки высококвалифицированных врачей, создана в 1925 г. Однако первый свод правил, регламентирующих ее организацию и отбор претендентов, появился лишь в феврале 1938 г. Согласно ему, в клиническую ординатуру принимались по конкурсу врачи до 35 лет с опытом работы не менее трех лет, и только в отдельных случаях зачислялись врачи из числа выпускников ВУЗов. Не все образовательные учреждения имели возможность организовать ординатуру, а только ведущие ВУЗы и научные организации, позже — институты усовершенствования врачей. Государственный институт экспериментальной эндокринологии и химии гормонов РСФСР получил право проведения ординатуры на своей базе одним из первых. Задача клинической ординатуры заключалась в глубоком освоении эндокринологии, принципов правильного клинического подхода к пациенту, овладении методами современного клинического исследования. Обучение исходно строилось на основании индивидуального плана, включавшего в себя теоретическую и лечебно-профилактическую работы с обязательной ежедневной проверкой сделанного на профессорских обходах, конференциях, индивидуальных собеседованиях. Ординаторы привлекались к составлению литературных обзоров, описанию отдельных случаев из практики, демонстрации пациентов на врачебных конференциях. Все вышеназванные обязанности ординаторов ставили своей целью расширение клинической эрудиции и мышления, воспитания навыков исследовательской работы, что было необходимо для дальнейшей врачебной деятельности и продолжения образовательного процесса в аспирантуре.

Профессор Николай Адольфович Шерешевский, ученик В.Д. Шервинского, в 1934 г. назначается на должность главы Всероссийского института экспериментальной эндокринологии и химии гормонов. Деятельность Николая Адольфовича была связана как с научно-исследовательской работой, так и с учебно-воспитательной. Объединение клиники и науки стало основой образовательной программы ординатуры по эндокринологии во Всероссийском институте экспериментальной эндокринологии и химии гормонов. Среди московских врачей того времени были широко известны «шерешевские консультационные приемы», в рамках которых разбирались самые тяжелые клинические случаи для диагностики и лечения по эндокринной патологии. Вот как описывают обучающиеся этот процесс: «В январе 1933 г. мы приступили к работе в клинике Института под непосредственным руководством проф. Шерешевского. Метод всестороннего обследования больных, разбора каждого из них в непосредственном присутствии и участии, последовательное выдвижение и изучение проблем, вытекающих из каждой категории заболеваний, и проработка наиболее злободневных из них на конференциях — обогащают наши знания, способствуют расширению врачебного кругозора и синтетическому подходу к предмету. Каждый из нас имеет свою палату в составе трех больных, которых он курирует самостоятельно под наблюдением старшего ассистента».

Екатерина Алексеевна Васюкова, ставшая в 1952 г. директором института, продолжила курс, взятый Николаем Адольфовичем. Ее клинические разборы и лекции отличались особой яркостью и глубиной содержания. Ею были написаны множественные сборники и руководства по эндокринологии. Проводимые семинарские занятия и клинические разборы тяжелых и интересных случаев заболеваний учили врачей формировать клиническое мышление.

Дальнейшее развитие образования перешло от клинической эндокринологии в сторону фундаментальной. Академиками АМН СССР, профессорами Николаем Алексеевичем Юдаевым и Юрием Александровичем Панковым, реорганизовывается структура института, строится и оснащается новейшим оборудованием экспериментальный корпус, организуются новые специализированные клинические и экспериментальные лаборатории, происходит подготовка фундаментально-ориентированных высококвалифицированных кадров и разрабатывается комплексная программа исследований в области эндокринологии в соответствии с задачами современной науки.

В 1989 г. директором института становится академик Иван Иванович Дедов, специалист с большим научным, клиническим и педагогическим опытом. Под его руководством вводится новая специальность «Диабетология» в качестве дополнительной подготовки эндокринологов при лечении пациентов с сахарным диабетом. Важным этапом руководства Ивана Ивановича становится введение обучения пациентов не только с сахарным диабетом, но и с другими патологиями эндокринной системы. В дальнейшем обучение пациентов внедряется на территории всей страны в рамках школ пациентов и федеральной программы «Борьба с сахарным диабетом», которая была инициирована и успешно реализована при его непосредственном участии и контроле. Новые междисциплинарные направления помощи пациентам, созданные И.И. Дедовым, плотно интегрировались не только в лечебный, но и в научный процесс, давая обучающимся бесценный клинический опыт.

В сентябре 2016 г. по приказу академика Ивана Ивановича Дедова на базе ФГБУ «НМИЦ эндокринологии» Минздрава России создается Институт высшего и дополнительного профессионального образования, директором которого назначается доктор медицинских наук Екатерина Александровна Пигарова. В Институте образования ГНЦ РФ ФГБУ «НМИЦ эндокринологии» Минздрава России создан фонд современной учебной литературы, авторами которой являются ведущие отечественные и зарубежные специалисты, выпускаются учебники, учебные пособия и монографии по эндокринологии. Помимо основных специальностей «Эндокринология» и «Детская эндокринология», с 2017 г. стартовало обучение по специальности «Диетология», которая, безусловно, является актуальнейшим направлением в современном мире и исконно близким к эндокринологии.

Педагогический и кадровый потенциал ГНЦ РФ ФГБУ «НМИЦ эндокринологии» Минздрава России уникален. На кафедрах трудятся настоящие профессионалы, обладающие исключительным педагогическим мастерством. Кафедрой эндокринологии заведует академик РАН Галина Афанасьевна Мельниченко, кафедрой диабетологии и диетологии — академик РАН Марина Владимировна Шестакова, кафедрой детской эндокринологии-диабетологии — академик РАН Валентина Александровна Петеркова.

Все кафедры проводят активную работу по созданию и внедрению инновационных подходов в медицинском образовании, формированию междисциплинарных оценочных процедур, использованию современных практикоориентированных образовательных технологий в системе подготовки кадров высшей квалификации.

В 2020 г., с приходом к руководству Центра члена-корреспондента РАН Натальи Георгиевны Мокрышевой, повышаются требования к ординаторам. Разрабатывается методика отбора обучающихся в ординатуре для дальнейшего прохождения обучения в передовых лабораториях Центра. Ежегодно более 30 ординаторов получают возможность освоить дополнительный курс по лабораторным методам исследования и лечения, поработать в лабораториях и защитить квалификационную работу. Система подготовки кадров включает также проводимые на ежегодной основе Конкурс молодых ученых, Конкурс научных проектов и Эндокринологический научный кружок. Все это позволяет сформировать у обучающихся важные научные компетенции, необходимые для дальнейшего планирования и выполнения научной работы. В 2020 г. создана кафедра персонализированной и трансляционной медицины, которую возглавила Наталья Георгиевна Мокрышева. Уникальной направленностью работы кафедры стало внедрение передовых технологий диагностики и лечения эндокринных заболеваний через современные образовательные технологии, а также формирование и развитие научно-исследовательских компетенций у обучающихся.

## Становление аспирантуры

Подготовка научных и научно-педагогических кадров через аспирантуру в нашей стране берет начало в 1925 г. Появление этой ступени послевузовского профессионального образования было вызвано острейшей нехваткой кадров высшей научной квалификации и неудовлетворительным функционированием существовавших на тот момент форм воспроизводства этих кадров. До начала 1930-х для регулирования сферы деятельности аспирантуры практически ничего не предпринималось: не было четких инструкций относительно форм и методов организации обучения в аспирантуре, руководства аспирантами, ответственности за их подготовку и т.д. Также не было ясности в вопросах критериев успешного прохождения аспирантуры и обязательной защиты диссертационной работы по завершению обучения.

Только с середины 1930 гг. в СССР стала формироваться система аспирантуры, создание диссертационных исследований и аттестация ученых. В Наркомздраве была создана специализированная комиссия по отбору аспирантов, которая включала выдающихся медиков под руководством Христиана Георгиевича Раковского. Для контроля за реализацией программ аспирантуры сформирована Центральная проверочная комиссия. Из отчетов комиссии 1935–1936 гг. понятно, что знание иностранного языка у аспирантов было крайне неудовлетворительно, многие плохо разбирались в основополагающих отраслях — физике, химии, биологии, биохимии, да подчас и в клинических дисциплинах.

13 января 1934 г. в свет выходит Постановление правительства «О подготовке научных и научно-педагогических работников», на основе которого была проведена проверка работы аспирантуры и вводились регулярные ежегодные проверки успеваемости аспирантов, четко определена образовательная составляющая аспирантской подготовки, и закреплена практика представления и защиты аспирантами диссертационной работы. Согласно этому постановлению аспиранты или соискатели ученой степени кандидата наук допускались к защите диссертации только после сдачи кандидатских экзаменов, которые включали экзамен по общей дисциплине, определяющей специальность аспиранта в целом, по другой дисциплине, соответствующей его более узкой специализации, а также по диалектическому и историческому материализму и иностранному языку.

План подготовки аспиранта был рассчитан, как и сейчас, на три года: на первом году обучения аспирант должен был заниматься изучением иностранных языков (не менее двух), марксистско-ленинской методологией, а также самостоятельной работой по специальности с консультацией научного руководителя и посещением лекций. На втором году к этому добавлялась педагогическая работа и начиналась работа над диссертацией. Третий год был в основном посвящен работе над диссертацией с консультацией научного руководителя, а также небольшой педагогической работе (порядка 20–25% рабочего времени аспиранта отводилось на работу с научным руководителем и 75–80% — на самостоятельную работу по теме исследования).

В 1938 г. секретарю центральной аспирантской комиссии Наркомздрава РСФСР от Николая Адольфовича Шерешевского поступает план работы для аспирантов. Было предложено прохождение аспирантуры цикловым методом: по 4 месяца работы в морфологической, патоморфологической и биохимической лабораториях, 2 месяца в лаборатории фармакологии и биологического контроля и полуторагодовалая работа в клиническом отделе института. На протяжении полутора лет аспирант был обязан выполнять определенные виды заданий: изучать клинику заболеваний эндокринной системы, самостоятельно вести эндокринных больных, детально обрабатывая каждый отдельный случай, изучать всю простую и сложную методику обследования эндокринного больного, прорабатывать разделы теоретической и клинической эндокринологии по соответствующей монографической литературе, обязательно изучать иностранные языки, выступать с докладами обзорного и клинического характера на конференциях института, вести самостоятельную научную работу под руководством директора клиники, принимать участие в коллективной научной работе клиники. Каждое полугодие аспирант был обязан сдавать специальный зачет по пройденному курсу и отчитываться о проделанной работе. По окончании аспирантуры необходимо было предоставить диссертацию на соискание ученой степени кандидата медицинских наук.

На протяжении следующих пятнадцати лет Институт экспериментальной эндокринологии создал квалифицированные научные кадры и практических работников, из которых многие получили ученые степени, организовал ряд курсов по усовершенствованию врачей. Народный комиссариат здравоохранения РСФСР, принимая во внимание все вышеуказанные заслуги Института, принял решение объявить благодарность Николаю Адольфовичу Шерешевскому и всему коллективу за проделанную работу по созданию и развитию советской эндокринологии.

Фундаментальные и клинические научные исследования в области эндокринологии при академике Иване Ивановиче Дедове вышли на новый уровень с выделением таких приоритетных направлений, как изучение нейроэндокринной системы в онто- и филогенезе, изучение геномных и постгеномных технологий, протеомных маркеров патогенеза болезней эндокринной системы, разработка и внедрение в клиническую практику новейших высокотехнологичных методов диагностики, лечения, профилактики и реабилитации больных эндокринопатиями, разработка генных, клеточных технологий и нанотехнологий в лечении гормонально-активных опухолей эндокринных желез, болезней гипоталамо-гипофизарной и репродуктивной систем, наследственных эндокринопатий детского возраста, проблем мужского и женского репродуктивного здоровья от рождения до мено- и андропаузы и многих других.

В 2020 г. под руководством академика РАН Ивана Ивановича Дедова и члена-корр. РАН Натальи Георгиевны Мокрышевой на базе «НМИЦ эндокринологии» создается уникальное научное подразделение — Национальный центр мирового уровня. В его структуре открывается множество фундаментальных лабораторий, которые дают возможность планирования и защиты диссертаций нового уровня и публикаций в ведущих журналах мира. Достижения центра были отмечены присвоением учреждению статуса Государственного научного центра в апреле 2022 г.

## Заключение

В настоящее время подготовка кадров для эндокринологической отрасли переживает существенный подъем, несмотря на выраженный дефицит кадров в медицине в целом, в том числе в области эндокринологии. Создание Института образования ГНЦ РФ ФГБУ «НМИЦ эндокринологии» Минздрава России позволило консолидировать современные образовательные технологии, расширить программы образования и подтвердить историческое значение Центра как флагмана эндокринологического образования и науки.

## Дополнительная информация

Источники финансирования. Работа выполнена по инициативе авторов без привлечения финансирования.

Конфликт интересов. Авторы декларируют отсутствие явных и потенциальных конфликтов интересов, связанных с содержанием настоящей статьи.

Участие авторов. Сумина С.В. — написание рукописи; Пигарова Е.А. — внесение существенных правок в рукопись; Дзеранова Л.К. — внесение существенных правок в рукопись; Кузьмин А.Г. — внесение существенных правок в рукопись.

Все авторы одобрили финальную версию статьи перед публикацией, выразили согласие нести ответственность за все аспекты работы, подразумевающую надлежащее изучение и решение вопросов, связанных с точностью или добросовестностью любой части работы.
